# Advances in Binders for Construction Materials

**DOI:** 10.3390/ma19020339

**Published:** 2026-01-14

**Authors:** Jorge Otero

**Affiliations:** 1 Research Group of Conservation of Cultural Heritage, Art and Conservation Department, Fine Arts Faculty, University of Barcelona, Pau Gargallo, 4, 08028 Barcelona, Spain; oteroj@ub.edu; Tel.: +34-934-03-40-51; 2 The Institute of Archaeology, University of Barcelona (IAUB), Montalegre 6-8, 08001 Barcelona, Spain

The global production of binders for construction materials exceeds 7.5 billion tons per year, contributing nearly 6% of total anthropogenic CO_2_ emissions [1]. Reducing this environmental footprint remains one of the most urgent challenges for the construction industry. In recent years, remarkable progress has been made toward developing alternative and more sustainable binders capable of replacing or complementing ordinary Portland cement while maintaining or even surpassing its performance [2].

Current worldwide research is driving this transformation by exploring innovative pathways to design low-carbon and high-performance binders that can serve as real alternatives to Portland cement. Among the most promising directions are calcium aluminate and calcium sulfoaluminate cements, alkali-activated binders, calcined clay–limestone systems, supersulfated cements, and nanomaterial-enhanced composites [3]. These new materials are reshaping how we understand hydration mechanisms, microstructure evolution, durability, and recyclability in both modern and historic contexts. In this context, the Special Issue Advances in Binders for Construction Materials has gathered a wealth of innovative research that exemplifies the diversity and dynamism of this field. Together, Volumes I and II have shown significant advances in both fundamental understanding and practical applications of new binder technologies, contributing to the evolution of sustainable construction science.

Across both volumes, contributions have spanned a broad range of topics—from novel materials and hydration mechanisms to performance optimization, durability, and digital modeling. The papers published across Volumes I and II have explored a wide range of advances in binder science. These include the development and characterization of alternative and low-carbon cements [4,5], enhancement of hydration kinetics using novel additives [6], microstructural and porosity analysis of cement pastes, integration of nanomaterials to improve mechanical and multifunctional properties, machine learning and artificial intelligence approaches to predict concrete and mortar performance, valorization of industrial wastes such as marble dust and marble powder for sustainable concrete formulations, investigations of debonding behavior and durability of fiber-reinforced cement-based repairs, and the fresh and hardened properties of ultra-high-performance concretes (UHPC), including applications in 3D-printed mortars. In addition to these experimental studies, the volumes also include comprehensive reviews that synthesize current knowledge on advanced binder applications, such as 3D printing of concrete and fly ash as a supplementary cementitious material. Among other additives and approaches, the volumes also cover studies on rice husk ash-based concrete, fly ash-based concrete, and plastering mortars modified with cellulose ether admixtures. Collectively, these articles illustrate the breadth and diversity of approaches taken by researchers to address both fundamental and applied challenges in sustainable binder development.

The works presented across both volumes have significantly deepened our understanding of the relationships between binder chemistry, hydration mechanisms, and performance under real service conditions. They have provided new insights into how alternative binders can achieve comparable or superior durability, mechanical strength, and functionality compared to traditional Portland cement systems. Equally important, the integration of nanotechnology, waste valorization, and machine learning has opened new avenues for designing and predicting binder behavior with unprecedented precision. These studies collectively demonstrate a paradigm shift from empirical optimization toward a scientifically grounded, data-driven, and performance-based approach to binder development. The research compiled here not only advances fundamental knowledge but also provides practical frameworks for industrial adoption, proving that sustainability and high performance are not mutually exclusive but can coexist through innovation and interdisciplinary collaboration.

Despite this progress, the path toward a fully decarbonized and circular construction industry remains challenging. Key gaps persist in understanding the long-term durability and environmental interactions of alternative binders under variable field conditions, as well as in standardizing methodologies for their production and characterization. The integration of life-cycle assessment (LCA) and multi-scale modeling into binder design remains underexplored and will be essential to evaluate true sustainability impacts. Moreover, the compatibility of new binders with existing infrastructure, particularly in repair and conservation contexts, requires further study to ensure safe and effective implementation. Future research must also expand beyond laboratory scales to pilot and full-scale applications, where issues of supply chains, cost, and regulatory acceptance become decisive. Addressing these challenges will require a concerted effort between academia, industry, and policymakers to transform scientific innovation into large-scale, tangible progress toward a carbon-neutral and resource-efficient construction ecosystem.
